# Computer-aided autism diagnosis based on visual attention models using eye tracking

**DOI:** 10.1038/s41598-021-89023-8

**Published:** 2021-05-12

**Authors:** Jessica S. Oliveira, Felipe O. Franco, Mirian C. Revers, Andréia F. Silva, Joana Portolese, Helena Brentani, Ariane Machado-Lima, Fátima L. S. Nunes

**Affiliations:** 1 School of Arts, Sciences and Humanities (EACH), University of Sao Paulo (USP), Sao Paulo, SP 03828-000 Brazil; 2 Department of Psychiatry, University of Sao Paulo’s School of Medicine (FMUSP), Sao Paulo, SP 05403-903 Brazil; 3 Interunit PostGraduate Program on Bioinformatics, Institute of Mathematics and Statistics (IME), University of Sao Paulo (USP), Sao Paulo, SP 05508-090 Brazil

**Keywords:** Computational biology and bioinformatics, Neuroscience, Computational neuroscience, Learning algorithms, Autism spectrum disorders, Computer science

## Abstract

An advantage of using eye tracking for diagnosis is that it is non-invasive and can be performed in individuals with different functional levels and ages. Computer/aided diagnosis using eye tracking data is commonly based on eye fixation points in some regions of interest (ROI) in an image. However, besides the need for every ROI demarcation in each image or video frame used in the experiment, the diversity of visual features contained in each ROI may compromise the characterization of visual attention in each group (case or control) and consequent diagnosis accuracy. Although some approaches use eye tracking signals for aiding diagnosis, it is still a challenge to identify frames of interest when videos are used as stimuli and to select relevant characteristics extracted from the videos. This is mainly observed in applications for autism spectrum disorder (ASD) diagnosis. To address these issues, the present paper proposes: (1) a computational method, integrating concepts of Visual Attention Model, Image Processing and Artificial Intelligence techniques for learning a model for each group (case and control) using eye tracking data, and (2) a supervised classifier that, using the learned models, performs the diagnosis. Although this approach is not disorder-specific, it was tested in the context of ASD diagnosis, obtaining an average of precision, recall and specificity of 90%, 69% and 93%, respectively.

## Introduction

Eye tracking is an approach explored by some computational systems to assist in the diagnosis of psychiatric disorders^1,2^. An example of disorder that is benefited from the eye tracking technology is the Autism Spectrum Disorder (ASD), a neurodevelopment disorder characterized by social interaction difficulties, as well as repetitive behaviors^3–5^. One of the early signs of ASD is the lack of eye contact^4,5^. This characteristic can be observed in toddlers as young as six months of age, regardless of the cultural environment the subject is in. Different studies, using a specific paradigm, certain regions of interest (ROIs) demarcated on each frame of a video, time and duration of fixation showed that ASD, compared to controls can be characterized by alterations in early precursor of social behavior as biological motion, human face preference, and joint attention.

Important results have been achieved using the total duration of gaze fixation in non-biological movements as a criterion to differentiate the subjects with and without ASD^6–10^. Pierce et al.^9^ differentiate groups with 21% of sensitivity and 98% of specificity. Wan et al^10^ discriminate groups with 86.5% of sensitivity and 83.8% of specificity. Shi et al.^6^ obtained an area under the ROC curve (AUC) of 0.86 with a sample composed of 33 children. Although, two drawbacks have been described in the ROI-based methods: (1) the need to demarcate each ROI on each frame of each video used in the experiments, and (2) information waste regarding which visual features of sub parts of an image had a more fixed gaze. Wang et al.^11^ showed the importance and contributions of including visual attention model (VAM) in ASD’s eye tracking studies.

The importance of image characteristics to VAM have been long recognize. To perform oriented goals, individuals must specifically allocate their attention, i.e., they must “select” some sensory inputs in detriment of others, translated as different neuronal firing. This is achieved by integrated bottom-up and top-down brain circuits. Bottom-up circuits are mostly based on image characteristics such as color, horizontal, vertical and geometry^12^. On the other hand, top down systems use an individual prior knowledge^13^ such as social rules, concepts learned and experienced selection models of what should be prioritized favoring the individual’s adaptability to the environment^14^, defined as semantic characteristics. The first computational VAM was developed by Koch et al.^15^, based on the Feature Integration Theory (FIT). Visual features such as color, orientation and intensity are extracted from the image of the scene. Then, all the feature maps are combined into a saliency topographic map. Finally, a cellular network Winner-Take-All is responsible to identify the most conspicuous location. Thus, processing only the fixation time or the fixation points in a pre-selected area does not allow to better understand the visual attention standard and its components, as suggested in some previous studies^6,11,16^.

Itti et al.^16^ made the first complete computational implementation of the Koch model, creating the most widely known and used model in the literature. Based on the implementation of Itti et al.^16^, other approaches were created, such as Borji et al.^17^ and Judd et al.^18^.

The models presented by Borji et al.^17^ and Judd et al.^18^ are based on pattern classification. They use supervised machine learning methods to learn the VAM using eye tracking data or pixels manually labeled as fixed or unfixed. Their models use images as inputs and extract around 26 features to form the feature vector used in the machine learning model. Their features are related to colors, orientation, intensity, steerable pyramids, horizon line, face, people and distance to the image center.

Approaches based on variations in visual attention standard, can establish different classes of individuals. Thus, a computational method can use this evidence to classify individuals into such classes. Each class can be efficiently modeled by a VAM, which can be defined as a description of the observed and/or predicted behavior of human visual attention^2,19^. Some recent works have been using VAMs to classify individuals using images^2,20,21^. Duan et al.^2^ state that VAMs applied to videos can contribute with more discoveries because the videos have temporal information.

This paper addresses some of the above-mentioned issues by proposing a machine learning approach to dispense the use of ROIs and develop a classifier based on VAMs learned for each group of individuals: ASD and Typical Development (TD). The main difference between this paper and those previously cited is the use of videos as input (instead of static images) to learn VAMs in order to aid ASD diagnosis using eye tracking signals. Videos can provide a more complete set of observations related to eye tracking but include some challenges to process. Additionally, our approach offers the possibility of using a video as stimuli for diagnosis different from that used in the VAM training. This difference represents some challenges for the model construction, whose solutions are the contributions of the present work. The proposed strategy could contribute not only in case/control comparison but also in the comparison of two disorders as ASD and Attention-Deficit / Hyperactivity Disorder (ADHD).

Thus, the main contributions of this paper are:

- an approach to infer two different VAMs—one for ASD individuals and the other for TD individuals—by using videos as stimuli and considering each group’s most relevant features;
- a technique to group frames of the video stimuli considering movement features;
- a method to classify an individual as ASD or TD, based on its adherence to the two VAMs previously cited, using any video as stimuli independently of the videos used for the VAM learning.

## Results and discussion

### Feature selection

Table 1 shows the 15 selected features by applying a Genetic Algorithm on the 28 original extracted features. As observed, no Red, Green, Blue color features were selected to classify the ASD visual attention. On the other hand, the feature related to the image center was only selected by the ASD group patients. These findings are in agreement with the results found by Wang et al.^11^, who realized that the ASD group had a greater focus on the center of the image, even when there was nothing in the center. We also tested the Relief algorithm to select features. However, the classification performance was worse than that using features selected by the Genetic Algorithm.

**Table 1:** Features selected by genetic algorithm for each category.

Features	# of ASD features	# of TD features
Steerable pyramids	3	4
Saliency toolbox: color, intensity, orientation and skin	4	4
RGB color	0	1
Horizon line	1	1
Presence of face	1	1
Presence of people	1	1
Distance to the frame center	1	0
Motion value	1	1
Presence of biological movement	1	0
Presence of geometrical movement	1	1
Distance to the side-specific scene center	1	1
Total	15	15

The features of the Saliency Toolbox related to the Itti model^22^ were selected for both groups, which provides indications of the biological relevance of such features, i.e., there is evidence that such features are important for visual attention for all humans in general, regardless of the presence of disorders such as ASD. For the TD group, the feature related to biological movement was not selected. This fact can be explained by the generic construction of the feature, that covers the whole region of the video that presents biological movement. Considering that the attention of the TD group is specifically more focused on the regions with people and faces (already covered by the other features), the biological movement does not reveal itself as a discriminant feature to obtain the VAM of the groups when the cited features set was used.

The features selected by the Genetic Algorithm are plausible with previous studies^6,9,11,23,24^ in terms of the relevance of the biological and geometric movement, image center, people and faces in the visual attention of individuals with ASD.

### Classification

**Figure 1:**
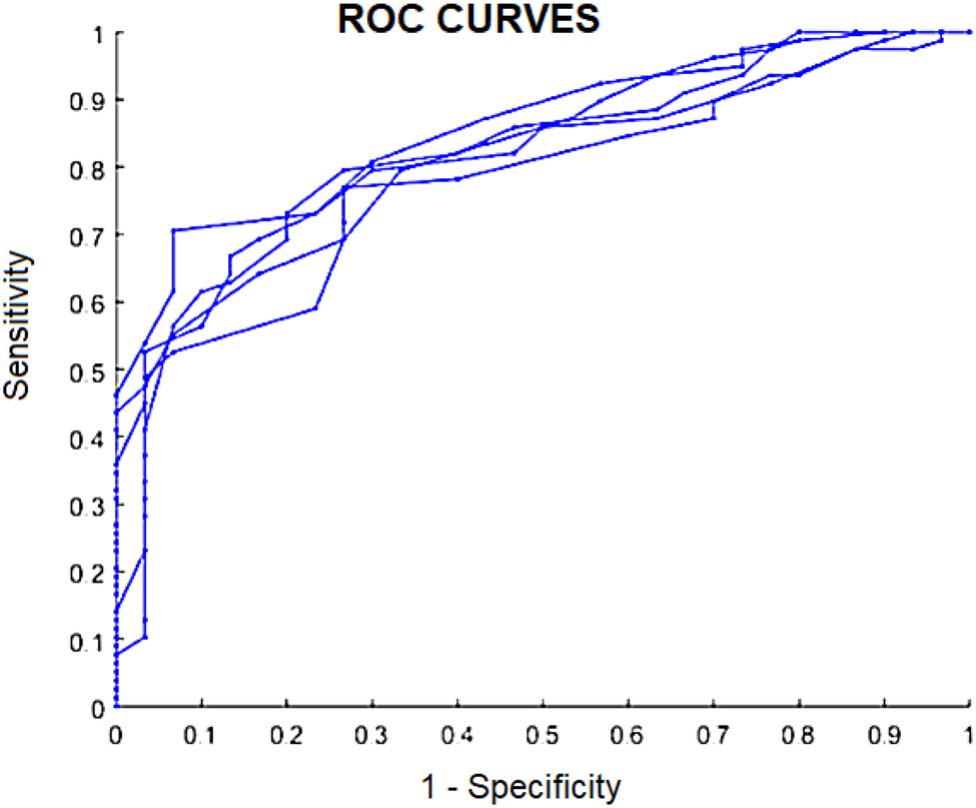
ROC Curves for Neural Networks with the features selected by the Genetic Algorithm. The 5 lines are the results of each of the 5-fold cross-validation rounds (this figure was built with MatLab 2015a version 8.5- www.mathworks.com/products/matlab.html^25^).

Figure 1 shows the ROC curves of the 5-fold cross-validation executions using the proposed method. Using the Youden method on the ROC curve we obtained a threshold of 28 frames, i.e., an individual was classified as belonging to the ASD class when 28 or more of her/his fixation maps agreed more with the ASD than with the TD saliency map. Using this threshold, the average results were 90% of precision, 93% of specificity and 69% of sensitivity/recall . Support Vector Machine (SVM) method was also evaluated as an alternative to Artificial Neural Networks (ANN) to learn the VAMs. However, the average AUC obtained by using ANN with Genetic Algorithm was 0.822, while the average AUC using SVM without feature selection was 0.775. In order to compare the approaches we evaluated, Table 2 presents the average AUC reached with each approach.

**Table 2:** Comparison of results of the evaluated approaches.

Classification algorithm	Feature selection algorithm	Average AUC (standard deviation)
SVM	None	0.775 (0.027)
SVM	Genetic algorithm	0.695 (0.023)
SVM	Relief	0.695 (0.042)
ANN	None	0.818 (0.053)
ANN	Genetic algorithm	0.822 (0.015)
ANN	Relief	0.782 (0.026)

In addition to the results obtained, showing the potential of the model itself, an advantage of using eye tracking for diagnosis is that it is non-invasive and can be performed in individuals with different functional levels and ages. Although there are papers that describe the classification of ASD based on eye tracking data^6,8,9,26^, the current proposal achieved this classification with AUC higher than most of the projects cited (Table 3), also using a heterogeneous dataset in terms of age, gender and CARS. In addition, analysis using VAMs avoids the need to demarcate regions of interest by a specialist, which can lead to data loss and bias.

**Table 3:** Comparison of results among related work.

Reference	Dataset	Average AUC
Chevallier et al.^26^	81 children (6–17 years)	0.71
Pierce et al.^9^	334 children (1–3 years)	0.71
Shi et al.^6^	33 children (4–6 years)	0.86
This work	106 children (3–18 years)	0.82

Several pro-cess steps have been modified from previous models^17,18^ to obtain better results, therefore they constitute contributions as well as topics for future research: an example is the grouping of frames using motion information, the pixel selection strategy, feature selection, similarity calculation and the classification process itself.

The classification proposal based on visual attention utilizing the above mentioned steps is innovative, not previously found in the literature. In addition to the entire proposed method for aiding ASD diagnosis, which presented promising results for the health area, the pipeline here defined constitutes a basis that can be reused or adapted to solve similar problems (where the attention can be indicative of the presence of the disorder) by computational approaches.

Finally, our approach can be applied using other visual stimuli, provided it is possible to extract the same features used. In addition, different stimuli can be used for VAM training and individual classification. This allows more flexibility to researchers of the health area and avoids the need of a database with specific stimuli. In the testing of the present article, we used the same videos for training and testing. Although it could be interpreted as contamination and biasing of learning, to circumvent this issue we did not use all the pixels in the VAM Learning phase. As described in section “Fixation map coordinate selection”, we select the 350 coordinates with the highest values to represent pixels of class 1 (related to fixations) and we also randomly select 350 pixels with zero fixation value to represent the class 0 (in which there was no fixation). We believe that this random selection approximates a scenario of usage of different videos, as long as these new videos use the same stimulus paradigm and have similar characteristics those used in this paper.

The approach presented in this paper processes eye tracking data to learn a supervised classifier based on VAMs. This approach achieved high performance (average precision of 90%) to classify individuals as belonging to the ASD or TD groups. Besides the social impact of the method, our approach offers a computational model that can be extended to be used as a tool for computer-based diagnosis of other disorders where the visual attention change is indicative of the presence of illness.

The method also brings some advances and presents research opportunities for the area of visual computing, since it presents different approaches in several stages of the developed method, such as: grouping of frames, selection of pixels, method of comparison between the fixation map and the saliency map, independence of stimuli, and the classification method itself.

A challenge to be overcome in this area is composing a robust dataset, since obtaining eye tracking signals with the respective evaluation of experts is not a trivial task. Thus, we intend to continue our dataset formation in order to make it available for the scientific community. We also intend to evaluate other machine learning techniques as well as to extract additional features, both aimed at improving the performance of the proposed approach.

## Material and methods

**Figure 2:**
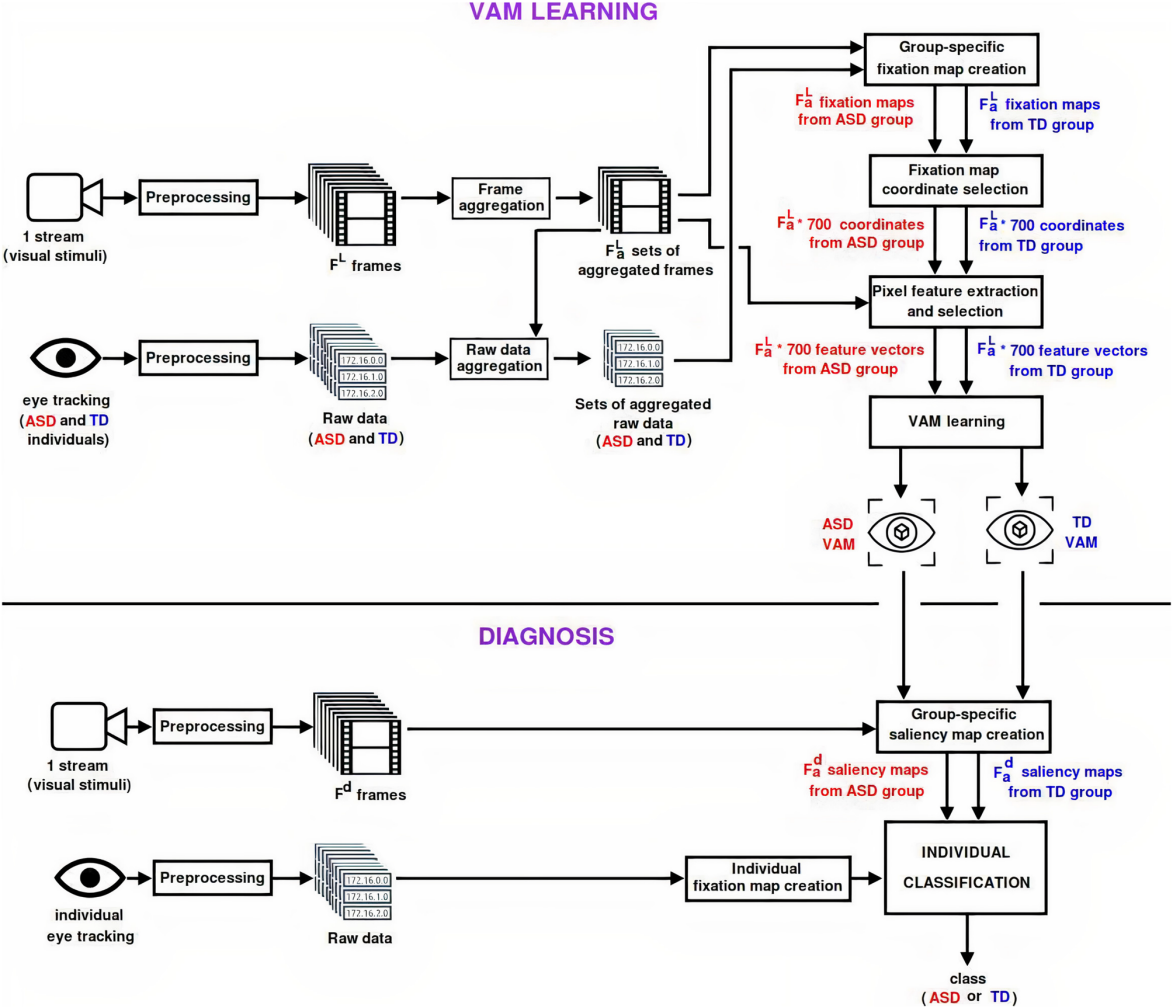
Overview of the entire process of the proposed model (this figure was built with XPaint version 2.9.10- https://directory.fsf.org/wiki/Xpaint^27^).

Figure 2 summarizes the entire method developed to classify a subject into ASD or TD class, composed of two phases: VAM learning and Diagnosis. The method considers two types of input data—a video used as stimulus and signals captured from an eye tracking—, which will be described in sections “Stimuli” and “Preprocessing”.

The VAM learning phase is responsible to process both the video used as stimulus and the eye tracking signals from the two groups (ASD and TD) to obtain a VAM model for each group. The frames of the video used as stimulus are submitted to a preprocessing step followed by a frame aggregation process. Similarly, the eye tracking signals are submitted to a preprocessing step followed by an aggregation process that follows the respective frame aggregations. The sets of aggregated frames and the sets of aggregated raw data are used together in the next steps: Group-specific fixation map creation, Fixation map coordinate selection, Pixel feature extraction and selection, and, finally the VAM learning. These eight steps are detailed in the subsections of section “VAM learning phase”. The Preprocessing step is described only once (since it is similar for both frames and raw data. Similarly, aggregation of frames and raw data is described together in the section “Frame and raw data aggregation”, also because the processing for both data types are the same.

The Diagnosis phase receive the same data from the first phase (video used as stimulus and eye tracking signals, not necessarily from the same stimulus used in the VAM learning phase) and, in addition, the learned ASD and TD VAMs. However, here the eye tracking signals are related to only an individual, who will be classified as belonging as ASD or TD class. For this, three steps are necessary: Group-specific saliency map creation, Individual fixation map creation, and, finally, individual classification. These three steps are detailed in the subsections of section “Diagnosis phase”.

It is important to highlight that, in the method evaluation, no information of subjects used for testing (“Diagnosis phase”) is used in the learning phase, once the cross-validation was performed over the subjects.

### Data acquisition

#### Ethical approval

The present study was approved by the Ethics Committee of the University of São Paulo, Brazil (protocol 57185516.9.0000.5390). All participants or their legal guardians signed an informed consent.

All procedures performed in this study, that involves human participants were in accordance with the ethical standards of the institutional and national research committee and with the 1964 Helsinki Declaration and its later amendments or comparable ethical standards.

The informed consent for publication of identifying information/images in an online open-access publication was obtained from the video participants.

#### Equipment and subjects

The eye tracking data was acquired using a Tobii Pro TX300 equipment^28^. Data from 106 subjects were collected to develop the model: 30 from the TD group (10 females and 20 males), and 76 from the ASD group (27 females and 49 males) All participants have age ranging from 3 to 18 years old.

The ASD subjects were recruited from the Psychiatry Institute, University of São Paulo School of Medicine (IPq-FMUSP), Brazil. The diagnoses were made based on the subject’s clinical evaluation by a child psychiatrist using the DSM-V (Diagnostic and Statistical Manual of Mental Disorders) criteria^3^ and ASD severity was measured using the Childhood Autism Rating Scale (CARS)^29^. CARS was also applied in TD subjects to confirm that they were out of the spectrum, with results below 30 points. Functional cognitive evaluation was performed by a trained neuropsychologist, using Wechsler Intelligence Scale for Children (WISC)^30^, Repetitive Behavior Scale (RBS)^31^, Vineland Adaptive Behavior Scales^32^ when possible. All clinical information of ASD individuals is available in Supplementary Material (Table S1).

ASD is a heterogeneous neurodevelopmental disorder and commonly co-occurs with other conditions such as psychiatric or neurological disorders^33^. Comorbidities vary according to different ages. Some comorbidities as Anxiety could be detected in 30-50% of ASD patients and attention-deficit/hyperactivity disorder (ADHD) in 40% of ASD infants^34^. Together with core symptoms, co-occurring emotional and behavioral problems are very often present and contribute to different ASD trajectories^35,36^. Considering these findings, individuals with comorbidities were not excluded from our study.

#### Stimuli

The visual stimuli for training the VAMs were built with the collaboration of experts. They consist of videos of about 6 s each, where each frame has spatial resolution of 1920 $\times$ 1080 pixels. In each video the computer screen was divided into two parts: one with biological movements, which presents the children’s interactions with each other, and another with geometric movements, which presents fractal movements.

**Figure 3:**
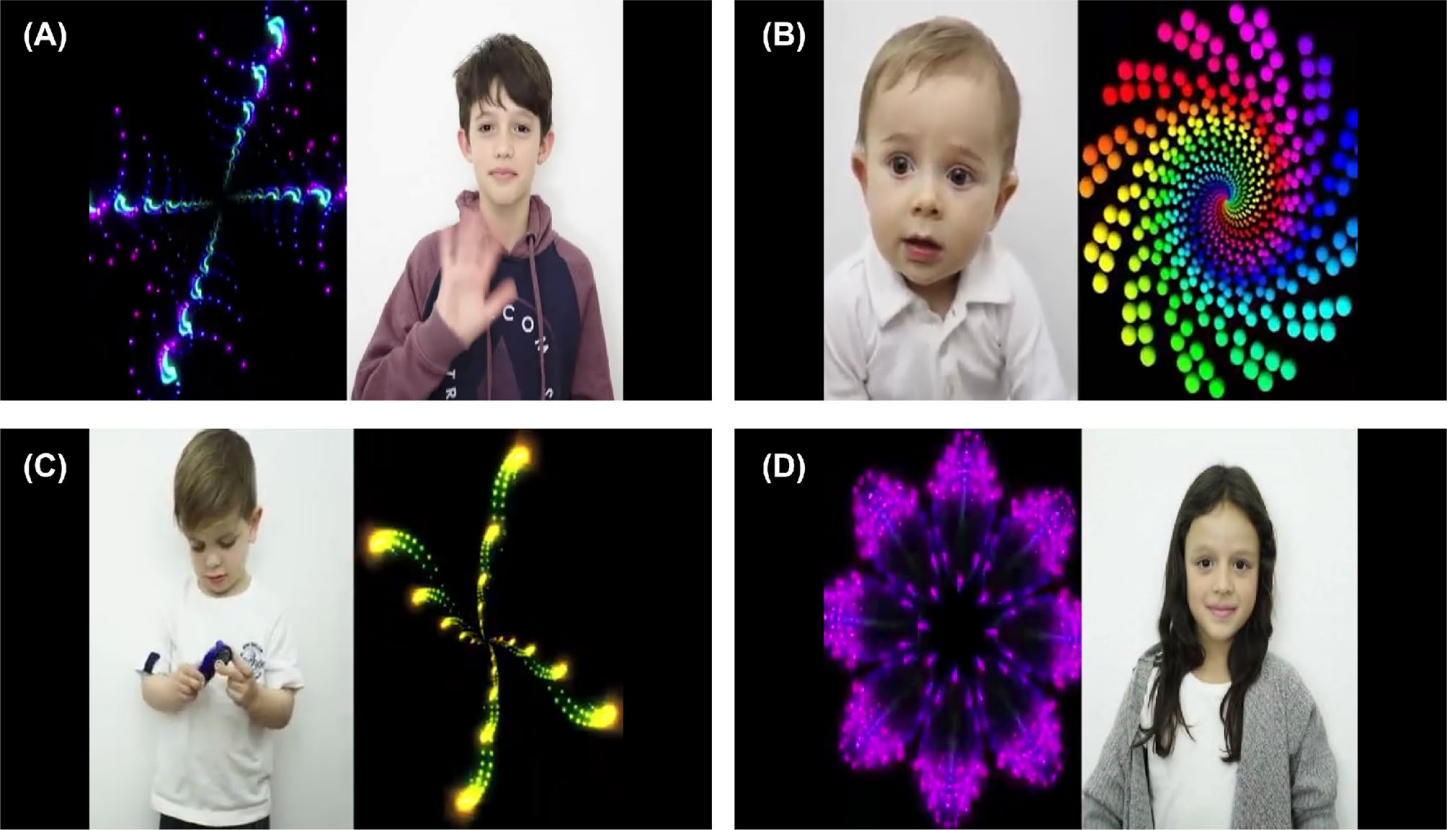
Example of frames of the video used as visual stimuli for training the Visual Attention Models (this figure was built in XPaint version 2.9.10- https://directory.fsf.org/wiki/Xpaint^27^).

Three videos with biological movements and three with geometric movements were combined, composing nine videos displayed sequentially, with total time of 54 s. Figure 3 presents some frames of the stimuli. The order and position of figures with biological and geometric movements are changed throughout the video in order to avoid conditioning of the subjects.

#### Protocol

A data acquisition protocol was defined, composed of three steps: participant positioning, equipment calibration and data acquisition.

In the first step, the subject was seated at a distance between 50 and 70 cm from the eye tracking monitor.

With the subject in a suitable position, a five-point eye tracking calibration was used. It shows an animated image at five different points on the screen. The subject was asked to follow the image with his/her gaze. Thus, the eye tracking device was able to recognize the eye position. In case of failure, the calibration was repeated. In case of a second failure, the subject was excluded from the experiment.

The acquisition was started after the calibration. During the entire session, an expert or caregiver was responsible for ensuring that the subject would remain seated and with his attention on the screen. Depending on the subject’s height it was necessary to sit him/her on the lap of an adult. In these cases, the adult used a blindfold in order to avoid influencing the signals acquired. All selected subjects had more than 80% of the total video time captured by the eye tracking equipment.

### Proposed method

The next subsections detail each step of the method presented in Fig. 2.

#### VAM learning phase

This section describes how the ASD and TD VAMs were learned. Each model is a binary classifier that given a pixel, with a set of features, it will output if this pixel will be fixed by the subject of a specific group or not.

Therefore, the objects used in the learning process of such models are the pixels that arise from the video processing, each one represented by a feature vector, described as follows. The classes considered were 1 (pixel was fixed) and 0 (pixel was not fixed).

##### Preprocessing

Initially the visual stimuli, which are in video format as previously described, were divided into frames. Then, a preprocessing was performed in each frame, which consisted of: removing the edges around the frame (black background, as can be seen in Fig. 3), resizing the frames to a resolution of 200 $\times$ 350 pixels and removing the transition frames between two videos (ten last frames of a video and ten first frames of the following video). In Fig. 2, $F^L$ is the number of frames that resulted from this process in this phase of VAM learning. The raw data provided by the eye tracker (pixel coordinates and timestamp) from ASD and TD individuals were also preprocessed in order to correspond to the same frames and regions.

##### Frame and raw data aggregation

The basis for the VAM learning is the information regarding which pixels were fixed by the subjects and which pixels were not. However, once the stimuli are videos, each single frame does not have enough fixation points to extract information. To circumvent this problem, we aggregated consecutive frames with a mean motion value among them of less than 0.33.

The concept of optical flow was used to compute the mean motion value. It is calculated by comparing a frame with the next one and returning a value of movement for each pixel. This value takes into consideration mainly the difference in intensity of a pixel in the current frame compared to the correspondent pixel in the next frame^37^. For the current frame, we sum all the values of motion of each pixel compared to the respective pixel in the next frame. Then, we divide the result by the total of pixels (7000). The final motion value is in interval $[0-1]$. If the final value is lower than 0.33 we aggregate the features and the frames themselves. corrFor this, the feature vector of each pixel of this frame aggregation consists of the mean value of the original values of the respective pixels. The resultant frame aggregation is compared to the next frame in order to verify if a new aggregation should be performed or not. The threshold was defined by analyzing visually the video used as stimulus to identify when images of two consecutive frames were nearly the same. We identified the average value of movement that allowed us to group consecutive frames whose variation in the pixels could indicate that no or little movement was detected. Using optical flow showed itself an efficient approach to do this task automatically. This value is directly related to the video. Thus, in case of using a different video, this value should be reviewed.

In Fig. 2, $F_{a}^L$ is the number of sets of aggregated frames that resulted from this process ($F_{a}^L < F^L$). For each set of aggregated frames, the corresponding raw data were also aggregated.

##### Creation of group-specific fixation map

**Figure 4:**
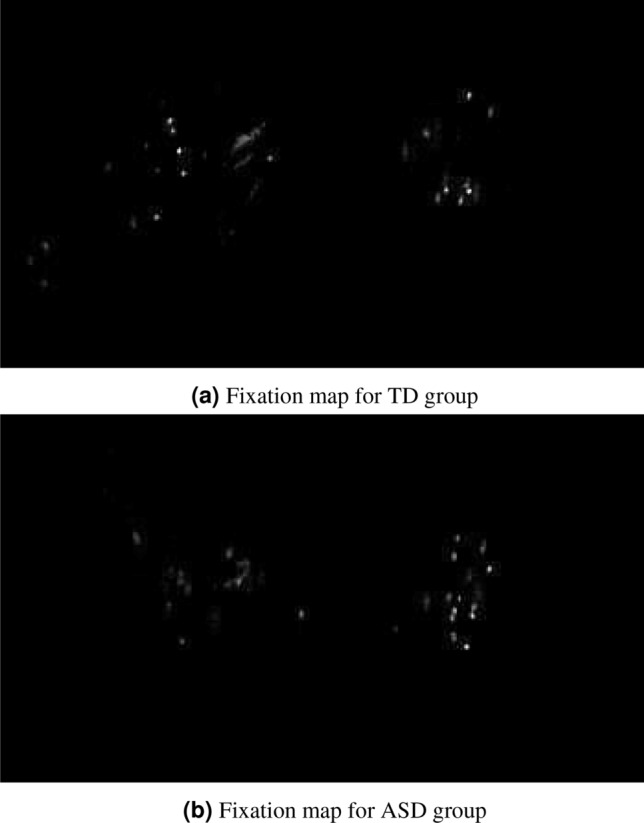
Example of fixation maps for a video frame that contains a scene of biological movement on the left side and a scene of geometric movement on the right side. The frame used for generating these maps are similar to frames B and C in Fig. 3 (this figure was built with MatLab 2015a version 8.5-www.mathworks.com/products/matlab.html^25^).

The sets of aggregated raw data from each group were used to create $F_{a}^L$ group-specific fixation maps. A fixation map is a matrix, with the same size of frames that compose the corresponding set of aggregated frames. Each position of this matrix has the number of gaze fixations in the respective coordinate. For each set of aggregated frames, two group-specific fixation maps were created summing up the number of fixations on the frames of all the subjects from a group (ASD or TD). In each map a Gaussian filter, with a kernel of size 5x5, was applied to smooth the fixations. This procedure generates a gray-level image that represents the fixation map where clearer cells indicate the positions that were most fixed by the group (Fig. 4).

##### Fixation map coordinate selection

The remaining processes of this phase aim to create the pixel feature vectors that will be used to train the ASD and TD models. These models are binary classifiers able to predict if a pixel will be fixed or not by the specific group, considering its features (section “Pixel feature extraction and selection”). The role of the fixation map coordinate selection is to define a balanced training sample for this purpose.

For each group-specific fixation map, the 350 coordinates with the highest values were selected to create the representative pixels of class 1 (in which there were fixations) and 350 pixels with zero fixation value were randomly selected as representative pixels of class 0 (in which there was no fixation). This process generated 700 coordinates for each fixation map from, summing up $F_{a}^L * 700$ coordinates for each group.

##### Pixel feature extraction and selection

The pixel feature extraction process is responsible for creating the 700 feature vectors from each group-specific fixation map, generating a total of $2* F_{a}^L * 700$ feature vectors. For each coordinate selected in the previous process (section “Fixation map coordinate selection”), the respective feature vector was composed of 28 features, each feature derived from all pixels presented in that coordinate in the aggregated frames corresponding to that group-specific fixation map. More specifically, each feature value of a specific coordinate was calculated by averaging the feature values of the pixels from the aggregated frames in that coordinate.

These 28 features were chosen based on the models most cited in the literature^17,18^. These models have defined the features considering studies on Biology and Psychology areas related to human visual attention. Moreover, some of these features, related to the face, presence of people and movement, are also relevant to the typical visual attention observed in individuals who belong to the ASD spectrum. We used the following features: 13 steerable pyramids with four scales and three orientations^38^; color, intensity, orientation, and the presence of skin (these four features were generated by the Saliency Toolbox^39^), three features representing the RGB (Red-Green-Blue) color channels; a feature indicating the presence of horizon line^18,40^ that was detected by using a mixture of linear regression trained with “gist” descriptor (a representation of an image in low dimension with information of the scene^41^); two features regarding the presence of faces and people, respectively^42^; one feature regarding the Euclidean distance from the current pixel to the central pixel of the screen and another feature with the Euclidean distance from the current pixel to the central pixel of the scene (the scene corresponds to the half of the screen where the current pixel is located); a feature indicating the amount of movement, calculated by optical flow^37^ (detailed in the section “Frame and raw data aggregation”); and the last two binary features indicating if the current pixel belongs to a biological or geometric scene.

After extracting the above mentioned features, we used a Genetic Algorithm^43^ to select the best features in distinguishing pixels from classes 0 and 1 for each group. The 15 best features (shown in section “Feature selection”) compose the feature vector used in the learning process of the VAM of each group.

##### VAM learning process

The $2* F_{a}^L *700$ feature vectors resulting from the previous process were used for learning the ASD and TD VAMs. For this learning we used a neural network with ten neurons in a single hidden layer and stop condition to achieve 1000 training cycles or error less than 1e−7. We used the binary cross-entropy as loss function, stochastic gradient descent as optimizer and a learning rate of 0.01. The activation functions were the sigmoid in the hidden layer and linear in the output layer. Each learned neural network (ASD or TD VAMs) is able to predict if a specific pixel, represented by its 15-feature vector, will be fixated by an individual from its specific group (ASD or TD) or not.

#### Diagnosis phase

This section describes how the ASD and TD VAMs were used in the diagnosis phase. The videos containing the stimuli used in this phase are independent from the videos used for the VAMs learning, which is a differential of our proposal. Since we work with features extracted from the pixels, which are used to learn the VAMs, any video with similar characteristics that we used (i.e., containing geometric and biological movement) can be used in this diagnosis phase. When different videos are used, they need to be preprocessed in the same way as the videos used for learning (section “Preprocessing”), generating $F^d$ frames for diagnosis. In this work we used the same stimuli, but with different frames for the learning and diagnosis phases, as described in section “Individual classification”.

##### Group-specific saliency map creation

A saliency map is a matrix, with the same dimension of the frame that contains in position (*i*, *j*) the probability of the pixel (*i*, *j*) of the frame to be fixed. However, our goal is to obtain binary saliency maps (in which each position is a 0 or 1 value) to compare them to the individual fixation maps (section “Individual fixation map creation”). Then, the ASD and TD VAMs, learned as described in the last section, can be applied in any stimuli to generate a corresponding binary saliency map based on the features of the frame pixels.

In this work, the two VAMs (ASD and TD) were applied to each pixel of each diagnosis frame, generating $F^d$ ASD binary saliency maps and $F^d$ TD binary saliency maps. Thus, the saliency map of a set of aggregated frames is a matrix where each position has a value 1, indicating the prediction that the respective pixel will be fixed by an individual of that group, or 0 otherwise.

##### Individual fixation map creation

In this step, the raw data captured by the eye tracker from the individual being analyzed is used to create a fixation map for each diagnosis frame. The fixation map of the subject is a matrix containing 0 in the positions related to the pixels that were not fixed and 1 in the positions of the pixels that were fixed by that subject. The procedure executed in this step generates $F^d$ fixation maps from that individual.

##### Individual classification

The classification process is responsible for answering to which group the individual belongs: ASD or TD. For this, the $F^d$ individual fixation maps (section “Individual fixation map creation”) are compared with the $F^d$ binary saliency maps from both groups.

For each diagnosis frame, the subject’s fixation map was compared to the binary saliency map generated for each group (section “Saliency map creation”). Given a position (*i*, *j*), a match occurs when the individual fixation map and the binary saliency map have the same value in this position or, in other words, when the model correctly predicts whether the pixel will or will not be fixed by that individual. That way, the number of matches between the two maps (individual and group) is considered a measure of similarity between them. The group of the saliency map (ASD or TD) that was most similar to the subject’s fixation map receives one vote to classify the subject.

As previously mentioned, our approach allows using any video for the diagnosis phase. In this work, instead of using different stimuli in the diagnosis phase, we used the same video. However, in order to simulate a different video, $F^d= 50$ frames from the original stimuli videos were removed from the VAM learning and used for this diagnosis phase. Each possible threshold of ASD votes needed to classify an individual to the ASD group leads to different classification performance measures, such as sensitivity and specificity. Then, a ROC (Receiver Operating Characteristic) curve can be created varying these threshold values.

The entire process (VAM learning and diagnosis, described in sections “VAM learning phase” and “Diagnosis phase” ) was repeated using a 5-fold cross-validation for the subjects. In each fold, the diagnosis phase was performed using data from 20% of the subjects and 50 diagnosis frames from the original stimuli video (composed of $F^L + F^d$ frames), whereas the VAM learning was performed using the remaining subjects and frames. Also, we used the ROC curve to apply the Youden^44^ method in order to calculate the best threshold of votes. The results of the five folds indicated that, from 50 diagnosis frames, the suitable threshold was 28 ASD votes to classify an individual to the ASD group.

## Supplementary Information

Supplementary Information
