# Supplementary material for: Computer-aided autism diagnosis based on visual attention models using eye tracking
Source: Sci Rep. 2021 May 12;11:10131. doi: 10.1038/s41598-021-89023-8 (PMC8115570; doi:10.1038/s41598-021-89023-8)
Supplement: Supplementary file 1 — Supplementary Information [file 41598_2021_89023_MOESM1_ESM.pdf]

Computer-aided autism diagnosis based on visual attention models using eye tracking

Jessica S. Oliveira[1], **Felipe O. Franco**[2,3], **Mirian C. Revers**[2], **Andréia F. Silva**[2], **Joana Portolese**[2], **Helena Brentani**[2], **Ariane Machado-Lima**[1], **and Fátima L. S. Nunes**[1,*]

[1]School of Arts, Sciences and Humanities (EACH), University of Sao Paulo (USP), Sao Paulo-SP, zip code 03828-000, Brazil
[2]Department of Psychiatry, University of Sao Paulo's School of Medicine (FMUSP), Sao Paulo-SP, zip code 05403-903, Brazil
[3]Interunit PostGraduate Program on Bioinformatics, Institute of Mathematics and Statistics (IME), University of Sao Paulo (USP), Sao Paulo-SP, zip code 05508-090, Brazil

Number	ID	Sex	Age	WISC	RBS	CARS	VTS	VC	VDL	VS	VL	Pharmacoterapy
1	1	M	10	154	6	33	-	-	-	-	-	Lisdexamfetamine
2	2	M	16	85	26	30	-	-	-	-	-	Risperidone, CBZ
3	3	M	6	132	9	30	95	116	100	97	72	Risperidone, CBZ
4	4	M	13	131	25	32.5	66	63	74	65	100	Methylphenidate
5	5	F	7	68	20	38	66	65	59	75	59	Not
6	6	M	10	-	-	-	-	-	-	-	-	-
7	7	M	15	48	18	33.5	52	45	61	49	78	Risperidone
8	9	M	15	54	21	39.5	54	53	54	55	56	Risperidone
9	10	M	4	-	9	31	-	-	-	-	-	Not
10	11	F	3	-	16	42	44	38	41	53	54	Risperidone, Vitamin-D
11	12	M	4	-	9	30	-	-	-	-	-	-
12	13	M	3	-	45	36.5	-	-	-	-	-	Not
13	14	M	15	62	53	40	64	62	65	70	91	Risperidone
14	15	F	8	62	22	34	64	69	69	59	84	Fluoxetine
15	16	F	9	62	1	32	85	74	102	85	91	Methylphenidate
16	17	F	4	-	51	35	57	52	64	61	61	
17	18	F	12	-	36	39	44	45	40	49	64	N-acetylcysteine, Lamotrigine
18	19	M	9	-	-	44	-	-	-	-	-	-
19	20	M	4	-	26	36	-	-	-	-	-	Not
20	21	M	6	-	38	34.5	61	57	66	61	70	Not
21	22	M	8		-	34	-	-	-	-	-	-
22	23	F	4	-	21	30	57	57	60	53	67	Not
23	24	M	12	88	14	31.5	77	81	73	85	97	Methylphenidate
24	25	F	11	126	20	31	76	81	78	75	84	Escitalopram
25	26	M	13	71	83	35.5	57	57	69	46	75	CBZ, Risperidone, Neuleptil
26	27	F	10	77	3	30.5	72	67	69	85	107	Neuleptil
27	28	M	11	-	12	30	60	56	63	64	104	Not
28	29	M	13	83	25	34	62	62	62	67	100	Risperidone, Methylphenidate
29	30	F	6	-	23	32	57	48	57	62	64	Not
30	31	M	-	-	-	-	-	-	-	-	-	-
31	32	M	11	100	17	30.5	64	65	69	64	104	Not
32	33	M	7	85	62	35	61	62	62	57	75	Aripiprazole, Neuleptil;
33	34	M	-	-	-	-	-	-	-	-	-	-
34	35	F	7	-	45	36	61	53	62	66	59	Not
35	36	M	10	146	20	30	67	65	71	71	84	OxCBZ
36	37	F	14	-	26	37.5	45	45	48	45	61	Not
37	38	F	9	77	56	30.5	66	81	61	62	67	Not
38	39	M	3	-	64	36	53	49	60	53	59	Not
39	40	M	8	115	30	30.5	64	69	63	64	70	Fluoxetine
40	41	F	4	-	38	32.5	60	57	64	61	67	Risperidone, Neuleptil;
41	42	M	11	80	7	35	63	62	66	64	88	Not
42	43	M	10	80	11	34	56	62	61	45	75	Fluoxetine, Methylphenidate
43	45	F	-	-	26	38	-	-	-	-	-	-
44	46	M	10	68	56	33	62	64	59	66	78	Haloperidol
45	48	M	9	77	11	36.5	55	47	61	57	78	Not
46	49	M	10	54	32	37	57	62	61	48	72	Not
47	50	M	10	71	30	34.5	62	64	59	68	107	Risperidone
48	51	F	7	-	22	45	50	50	51	38	91	Clonidine
49	52	F	11	-	45	41	47	54	51	38	91	Sertraline, Topiramate
50	53	M	11	97	30	31	63	69	62	62	88	Risperidone, Omega-3
51	54	M	5	-	21	37	57	49	66	61	64	Not
52	55	M	12	-	18	42	39	38	40	45	59	Fluoxetine
53	56	F	7	132	12	30.5	79	86	76	80	78	Risperidone, Methylphenidate
54	57	M	4	71	41	37	65	67	68	66	84	Clobazam, CBZ, Risperidone
55	58	M	8	-	59	35.5	61	62	59	61	57	Fluoxetine, Neuleptil
56	59	M	11	80	19	32	60	61	57	66	88	Not
57	60	M	9	87	17	33	78	67	97	76	104	Risperidone
58	63	M	9	-	25	31	54	53	55	53	81	Not

Number	ID	Sex	Age	WISC	RBS	CARS	VTS	VC	VDL	VS	VL	Pharmacoterapy
59	64	F	8	-	15	33.5	58	56	68	51	67	Risperidone, Topiramate
60	65	F	12	94	49	32.5	69	74	74	64	81	Risperidone, MEL
61	66	M	11	-	16	40.5	51	48	51	53	67	CBZ
62	67	M	11	83	40	37	68	64	83	62	100	Risperidone
63	68	M	10	-	80	44.5	41	38	51	38	59	Risperidone
64	69	M	8	117	42	30.5	64	65	66	66	84	Risperidone
65	71	F	10	97	67	38.5	60	61	61	62	94	Risperidone, Methylphenidate
66	72	F	3	-	18	32.5	64	67	66	65	72	Not
67	73	F	11	-	26	42	35	34	43	36	56	Risperidone, Sertraline
68	74	F	5	-	-	-	-	-	-	-	-	-
69	75	F	5	-	30	39	58	69	62	51	61	MEL
70	77	F	4	-	58	38	69	69	83	77	59	Risperidone
71	78	F	5	-	39	35.5	55	57	55	59	61	MEL
72	107	M	18	-	-	-	-	-	-	-	-	-
73	108	M	18	-	-	-	-	-	-	-	-	-
74	109	M	6	-	-	-	-	-	-	-	-	-
75	110	M	10	-	-	-	-	-	-	-	-	Fluoxetine, VPA, MEL, Neuleptil
76	111	M	3	-	-	-	-	-	-	-	-	-
Mean		-	8.8	89	30	35	60.5	60.6	63.8	61	77	-
SD		-	3.7	27.1	18.7	4	10.7	13.4	12.6	12.6	15.5	-
Median		-	9	83	25	34.5	61	62	62	62	75	-

Supplementary Table S1. Clinical information of ASD individuals. Age is expressed in years. Abbreviations: ID, Project Identification Number; WISC, Wechsler Intelligence Scale for Children; RBS, Repetitive Behavior Scale; CARS, Childhood Autism Rating Scale; VTS, Vineland Total Score; VC, Vineland Communication; VDL, Vineland Daily Living; VS, Vineland Socialization; VL, Vineland Locomotion; CBZ, Carbamazepine; VPA, Valproic Acid; MEL, Melatonin.
